# A Generative Pretrained Transformer (GPT)–Powered Chatbot as a Simulated Patient to Practice History Taking: Prospective, Mixed Methods Study

**DOI:** 10.2196/53961

**Published:** 2024-01-16

**Authors:** Friederike Holderried, Christian Stegemann–Philipps, Lea Herschbach, Julia-Astrid Moldt, Andrew Nevins, Jan Griewatz, Martin Holderried, Anne Herrmann-Werner, Teresa Festl-Wietek, Moritz Mahling

**Affiliations:** 1 Tübingen Institute for Medical Education Eberhard Karls University Tübingen Germany; 2 Division of Infectious Diseases Stanford University School of Medicine Stanford, CA United States; 3 Department of Medical Development, Process and Quality Management University Hospital Tübingen Tübingen Germany; 4 Department of Diabetology, Endocrinology, Nephrology, Section of Nephrology and Hypertension University Hospital Tübingen Tübingen Germany

**Keywords:** simulated patient, GPT, generative pretrained transformer, ChatGPT, history taking, medical education, documentation, history, simulated, simulation, simulations, NLP, natural language processing, artificial intelligence, interactive, chatbot, chatbots, conversational agent, conversational agents, answer, answers, response, responses, human computer, human machine, usability, satisfaction

## Abstract

**Background:**

Communication is a core competency of medical professionals and of utmost importance for patient safety. Although medical curricula emphasize communication training, traditional formats, such as real or simulated patient interactions, can present psychological stress and are limited in repetition. The recent emergence of large language models (LLMs), such as generative pretrained transformer (GPT), offers an opportunity to overcome these restrictions

**Objective:**

The aim of this study was to explore the feasibility of a GPT-driven chatbot to practice history taking, one of the core competencies of communication.

**Methods:**

We developed an interactive chatbot interface using GPT-3.5 and a specific prompt including a chatbot-optimized illness script and a behavioral component. Following a mixed methods approach, we invited medical students to voluntarily practice history taking. To determine whether GPT provides suitable answers as a simulated patient, the conversations were recorded and analyzed using quantitative and qualitative approaches. We analyzed the extent to which the questions and answers aligned with the provided script, as well as the medical plausibility of the answers. Finally, the students filled out the Chatbot Usability Questionnaire (CUQ).

**Results:**

A total of 28 students practiced with our chatbot (mean age 23.4, SD 2.9 years). We recorded a total of 826 question-answer pairs (QAPs), with a median of 27.5 QAPs per conversation and 94.7% (n=782) pertaining to history taking. When questions were explicitly covered by the script (n=502, 60.3%), the GPT-provided answers were mostly based on explicit script information (n=471, 94.4%). For questions not covered by the script (n=195, 23.4%), the GPT answers used 56.4% (n=110) fictitious information. Regarding plausibility, 842 (97.9%) of 860 QAPs were rated as plausible. Of the 14 (2.1%) implausible answers, GPT provided answers rated as socially desirable, leaving role identity, ignoring script information, illogical reasoning, and calculation error. Despite these results, the CUQ revealed an overall positive user experience (77/100 points).

**Conclusions:**

Our data showed that LLMs, such as GPT, can provide a simulated patient experience and yield a good user experience and a majority of plausible answers. Our analysis revealed that GPT-provided answers use either explicit script information or are based on available information, which can be understood as abductive reasoning. Although rare, the GPT-based chatbot provides implausible information in some instances, with the major tendency being socially desirable instead of medically plausible information.

## Introduction

Communication is one of the core competencies of health care professionals [1,2]. In the medical context, communication serves multiple functions, including relationship building, information gathering, and decision-making [3]. The ability to communicate with patients is crucial for their health outcomes [4,5]. Furthermore, inadequate communication can result in missed diagnostic opportunities and thus poses a hazard to patient safety [6,7]. Consequently, medical curricula worldwide incorporate either dedicated communication courses or a communication curriculum, depending on the level of curricular integration [8-10]. Formats that allow for the acquisition of communication competencies include theoretical lessons, peer-assisted learning, learning with simulation patients, and learning with real patients [11,12].

In this study, we assessed the potential of large language models (LLMs), such as generative pretrained transformer (GPT), in enhancing communication training. One key skill in medical communication is history taking, which is required in almost all medical fields to make a correct diagnosis and initiate treatment [13]. This learning objective typically starts with taking a systematic history (ie, assessing the history regarding all relevant body functions and organ systems). To practice history taking, the learner is required to have an interactive encounter [14], and courses frequently rely on simulated or real patients [15]. These formats are associated with high costs and a high organizational effort, however, which shortens the time available to acquire these skills. These restrictions often do not allow all students to interactively practice a skill or practice for more than 1 repetition [16]. Furthermore, learning in these settings often occurs supervised by the patient and peer group, thereby impacting performance and possibly inhibiting rather shy students from using the learning opportunity [17,18].

Chatbots offer a significant potential to overcome these restrictions, thereby enhancing the utility thereof in health care and medical education settings. Chatbots have thus become valuable tools in health care; their nonjudgmental and easily accessible nature makes them particularly well suited for responding to patient inquiries and concerns [19,20]. The use of chatbots in medical education offers equally promising opportunities. In particular, chatbots are of interest tool-wise in the area of virtual patients [21,22].

The advance of chatbots is significantly supported by the developments of LLMs, such as GPT, which progressed considerably in 2022 [23]. This progress in artificial intelligence (AI) technology opens up new horizons for innovative, cost-effective, and accessible learning methods [24,25]. GPT has performed surprisingly well regarding medical knowledge, including board exams [26-28]. The combination of excellent language skills and medical knowledge predispose GPT to perform as a chatbot. Moreover, LLMs allow for unsupervised and repeated learning, thereby enabling all students to learn for as long as it is needed. However, LLMs, such as GPT, are language models using a next-word prediction paradigm [29] and are thus prone to “hallucinations” (ie. producing nonsensical content) [30]*.* Moreover, LLMs are also known to occasionally escape prompts.

Chatbots have been used in medical education before the broader application of LLMs [31]. However, these virtual simulated patients did not reach human performance in terms of language expression and dynamics [31]. Although chatbots to practice history taking have been developed based on pre-LLM technology [32], it is unknown whether and how LLMs, such as GPT, can be used as a simulated patient to acquire communication skills. To investigate the previously uncharted potential of GPT as a simulated patient, we conducted a mixed methods study. Here, we present our analysis of GPT capabilities, as a chatbot as well as an improved version of an AI-optimized illness script.

## Methods

### Study Outline

First, we developed an illness script [33] that contained relevant medical information from a fictitious patient and a prompt to make GPT-3.5 (OpenAI) act as a simulated patient. We introduced the chatbot to medical students through a web interface, allowing them to voluntarily practice their history-taking skills. The conversations were recorded and systematically analyzed to explore the conversations with the GPT-powered chatbot. We focused on feasibility and usability and performed a quality assessment of GPT’s text output.

### Setting and Participants

During a large-scale skill-refreshing event with participants from all our faculty, students were invited to voluntarily participate in our investigation. After they provided informed consent, students were provided with a laptop on which the interface was ready to use. After entering demographic information, students could chat for as long as they felt necessary.

Since our participants were native German speakers, we conducted all interactions with GPT in German and later translated the data and screenshots into English for this paper.

### Chat Platform

**Figure 1:**
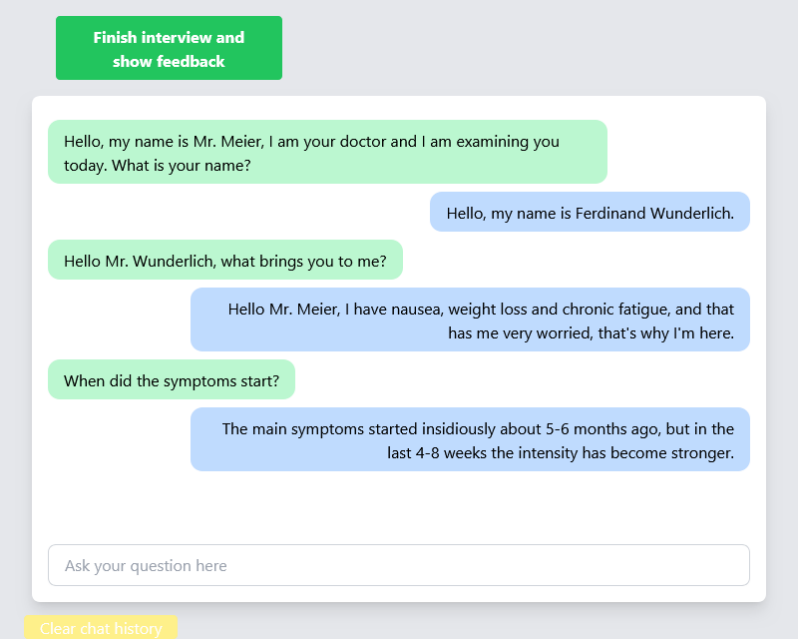
Screenshot of self-developed web interface.

To enable the interaction between students and GPT, we created a chat interface through which the students could post written questions to a virtual patient and receive written answers (Figure 1). This interface enabled us to guide user input and send system messages to GPT. The system was developed as a local HTML file. It used JavaScript code for processing and *tailwindcss* for layout. We called the OpenAI application programming interface (API) using the JavaScript Fetch API and making calls to OpenAI’s chat/completions endpoint using *gpt-3.5-turbo*. Model parameters were left at default settings. The complete chat history for each user input up to that point was sent to the model. At the conclusion of the conversation, the full chat history was saved to a text file for further processing.

### Prompt Development

Next, we developed prompts that were needed to make GPT act as a simulated patient. The prompts were designed to guide GPT’s behavior and ensure it provided medically accurate and relevant responses. Presented in detail next, our prompt included a chatbot-optimized illness script as well as a behavioral instruction prompt.

#### Chatbot-Optimized Illness Script With a Medical Case

We developed a fictitious medical case in a format that could be posted to GPT. As our learning objective was to take a systematic history, we intended to provide all required details. A short version with some information about the case is presented in Table 1, and the full case is provided as Multimedia Appendix 1.

**Table 1:** Illness script “Nausea, weight loss, and chronic fatigue” (shortened version).

Variable	Details
Patient details	Ferdinand Wunderlich, 48 years of age Occupation: administrative employee in the finance department of a municipal hospital Personal life: overweight, previously tried diets unsuccessfully; enjoys family time, has two sons aged 8 and 6 years; not physically active Initial consultation with a new general practitioner
Medical concerns	Presenting with nausea (especially after large meals), significant weight loss (10 kg in 6 weeks), and chronic fatigue Muscle cramps mainly in the legs and frequent at night Mental fatigue, with forgetfulness at work Has felt run down and tired for about 5-6 months, with symptoms intensifying in the past 4-8 weeks Feels severely limited by his current condition
Accompanying symptoms	Multiple minor infections recently Episodes of dizziness (ie, light-headedness) occurring 1-2 times daily Dry skin Increased thirst (drinks about 4-5 L of water daily) and frequent urination day and night
Medical history	Known hypertension, currently on blood pressure medication (Hygroton 50 mg and ramipril 5 mg) Shortness of breath during exertion Fatty liver diagnosed 3 years ago Right inguinal hernia treated surgically 3 years ago Mild constipation Allergic to penicillin since childhood Previously smoked for 4 years in his twenties Consumes beer occasionally (1-2 times a week)
Family history	Father died of a heart attack Mother died at 79 years of age and had diabetes later in life Brother diagnosed with colon cancer

#### Behavioral Prompt

In addition to the required medical information, it was necessary to instruct GPT to behave as a simulated patient, which is why we developed a behavioral prompt. To achieve this, we used our custom interface to test the answers provided by GPT by conducting the interviews ourselves. Where we noticed a failure to stick to the provided medical information, we tried to improve the manner in which the information was presented. For improvements to the prompt, we relied on our experience as well as the advice and model explanation provided by OpenAI [34].

During the iterative process of prompt development, 2 areas of improvement were evident: the role-play aspect (ie, that GPT sticks to the role as a patient) and the medical aspect (ie, that GPT provides answers as close as possible to the given information, while sounding human).

Regarding role-play, the model often struggled to maintain its assigned role, especially during discussions of potentially serious medical issues. We had little success with providing details of the role or simply reinforcing that the goal was to impersonate a patient. Instead, we found the most helpful tweak was adding “patient name:” at the end of any user input, where “patient name” would be replaced by the name specific to each case. This resulted in GPT generating a continuation of “patient name:,” making it more probable that the LLM would actually produce a sensible utterance by the patient. Other tweaks were to begin the initial system message with the patient’s name and continue to use this name to “address” GPT in this manner. We also instructed the model to not assist the user in this setting but to impersonate the patient, although we found this to have a much smaller effect than the other changes. Notably, the model was instructed to provide short answers to reduce reading times.

We provided GPT with the case description, preceded by instructions to use this information for answering medical questions. We also provided a list of all categories the student should ask about in the interview. The list contained possible answers and information for each category; for this list, we also included a statement about its format (ie, we explicitly stated that “[the list] will have the form ‘category’: ‘information or possible answer if asked’”). In general, surrounding factual information with an explicit description of its content and format increased the reliability of using that information.

It is important to note that formatting was also important, as the model sometimes picked up patterns in formatting in its own answers. Since the medical information was first produced with common text editing software, a simple copy and paste into our system also copied large amounts of formatting, such as indents, bullet points, or whitespace. Cleaning this formatting from the prompt helped the model avoid repeating these patterns in the output.

In a similar way, we tried to give more structure to the prompt by using special delimiter statements, such as “===DIALOGUESTART.” These were intended to help the model switch from reading in medical information to impersonating a patient. However, our approach was not successful, as the model started to repeat such patterns in its output, sometimes even initiating further switches, for example, by inserting “===DIALOGUEEND” itself. We had more success in achieving the desired behavior using structuring with explicit descriptions in natural language, as described before.

#### Full Prompt

The full prompt including both aforementioned parts is presented in Textbox 1.

**Prompt sent to the generative pretrained transformer (GPT) application programming interface (API) in JavaScript Object Notation (JSON) format. The prompt consists of a behavioral instruction prompt and the first user message. Further dialogue was appended during the interview.**

{

“role”: “system”,

“content”: “Hello Mr. Wunderlich, in the following you will assume the role of an acting patient. You will not assist the user, but answer questions based on the following information: Your name is Ferdinand Wunderlich,

[… Further Case Information ….]

Here is some more information on your complaints, Mr. Wunderlich. These are in the form of ‘Category’: ‘Information or possible answer on request’

Chief complaint, if applicable, with: Nausea and weight loss (most recently 10 kg in 6 weeks) Chronic fatigue, exhaustion and lack of drive

Localization and spread: The muscle cramps occur mainly in the legs.

[ … Further details (see illness script) …]

In the following, you will take the role of Ferdinand Wunderlich, […], that is, you will answer as Ferdinand Wunderlich. Try to keep your answers rather short.”

},

{

“role”: “user”,

“content”: “[USER INPUT]

“Ferdinand Wunderlich:”

}

### Qualitative Analysis

To gain a more profound understanding of the conversation between chatbot and participant, we conducted a qualitative analysis (ie, the Braun-Clarke inductive approach [35]) of the question-answer pairs (QAPs). All QAPs were thus aggregated and stored in a Microsoft Excel (version 16.0.10394.20022) sheet. For each question and answer, the word count was automatically analyzed using an Excel formula. Each QAP was assessed by 1 rater (author FH) for the aspects listed in Table 2. In equivocal cases, the items were discussed with another rater (author MM) until consensus was reached.

**Table 2:** Aspects, categories, and text examples used for rating.

Aspect and categories		Example(s)
**Conversation part**		
	Greeting/introduction	Student: “Good day, my name is Mrs Müller, and I am your attending physician. What brings you to me today?” GPT^a^: “Good day, Mrs Müller. I have been experiencing nausea, weight loss, chronic fatigue, and lethargy for some time and would like to find out what could be causing it.”
	Medical history	Student: “How much are you limited in your daily life?” GPT: “I am now severely restricted. After a long day at work, I am incapable of doing anything in the evening, and I also notice that I keep forgetting things at work. This way, I cannot manage either my work or the tasks at home in the family.” Student: “I will ask you briefly about a few other points that could be relevant. Do you have headaches?” GPT: “No, I do not have headaches.”
	Goodbye/outlook	Student: “Okay. We will now conduct some examinations. Thank you for your responses.” GPT: “Thank you for the questions and your attention. I hope we can find the cause of my complaints and a suitable treatment option.”
	Interactive exchange	Student: “I will discuss this with my boss and let you know tomorrow.” GPT: “Okay, that sounds good. Thank you for your help. I look forward to your feedback tomorrow.”
**Student’s question covered by the illness script**		
	Question not covered by the script	Student: “Have you been on vacation recently?” Student: “I imagine you sit a lot at your work. Do you have any back pain?”
	Question indirectly/partially covered by the script	Student: “Are your lymph nodes enlarged?” Student: “Do you have a history of thyroid disease?”
	Question explicitly covered by the script	Student: “What medications are you taking and at what dosage?”
	Statement (no question)	Student: “Thank you for the conversation! I would now like to conduct a physical examination and initiate further diagnostics.”
**GPT’s answer uses the illness script**		
	Answer based on explicit script information	GPT: “For several months, I have been increasingly struggling with chronic fatigue and a lack of drive. The weight loss of 10 kg in the past 6 weeks has additionally worried me.”
	Answer based on implicit script information	GPT: “No, no laboratory diagnostics have been carried out so far.”
	Answer based on fictitious information	GPT: “No, due to the current situation around the COVID-19 pandemic, I have not traveled recently.”
**Answer medically plausible (regarding case and illness script)**		
	+2: Answer plausible, highly specific for case	Student: “Okay, how much do you drink in a day?” GPT: “I currently drink about 4-5 L a day, I have a noticeably increased thirst.” Student: “All right, you also mentioned weight loss. How many kilograms are we talking about?” GPT: “I have lost about 10 kg in the past 6 weeks.”
	+1: Answer plausible, relevant for case	Student: “Do you have any accompanying pain?” GPT: “So far, I have not noticed any particular pains, except maybe occasional muscle cramps in my legs.”
	0: Answer plausible, not case specific	Student: “Have you ever had surgery?” GPT: “Yes, I had laparoscopic surgery for a right inguinal hernia 3 years ago.”
	−1: Answer rather implausible	GPT: “I do not follow any specific diet or way of eating. In principle, I eat quite balanced.” GPT: “I used to exercise regularly, but in the past few months, I have been unable to do any sports due to my complaints.” *Comment: Both statements do not fully align with the predefined role of a patient with long-standing obesity,* *but they show aspects of social desirability.*
	−2: Answer very implausible	GPT: “On average, I drink about 10-12 L of fluids per week. This includes water, tea, and occasionally other drinks, such as juice spritzers.” *Comment: Here, ChatGPT fails to convert the specified daily fluid intake into a weekly intake. Since polydipsia is an important symptom of hyperglycemia, this statement was rated as very implausible.*
	Not applicable	*Comment: For QAPs^b^ with empty answers due to technical reasons.*

^a^GPT: generative pretrained transformer.

^b^QAP: question-answer pair.

### Chatbot Usability Questionnaire

To assess how our participants perceived the simulated patient, we used the Chatbot Usability Questionnaire (CUQ) [36]. This 16‑item questionnaire measures the personality, user experience, error management, and onboarding of a chatbot and has recently been validated [37]. After ending the chatbot sessions, our participants were asked to fill out a written version of the CUQ, and the CUQ score was calculated using the tool provided by the authors [38].

### Quantitative Analysis

Statistical analysis and figure generation were performed with R statistical software (version 4.3.1; R Foundation for Statistical Computing) [39]. For the CUQ, we provided relative numbers of Likert categories. For counts, we reported the total (n) as well as percentages. Numerical data were inspected for normal distribution and provided as the mean and SD. If a Gaussian distribution could not be assumed, median and 25%-75% quartiles (Q25-Q75) were provided. We used the Spearman correlation coefficient to check for correlations, considering *P*<.05 as statistically significant.

### Ethical Considerations

The study was approved by the Ethics Committee of the Faculty of Medicine at University Hospital Tübingen (385/2023A). Data were kept anonymous and were not associated with students. Although the participant got an opportunity to use the chatbot without providing consent that the data could be used for our study, all students consented that their data could be used.

## Results

### Demographic Data of Participants

A total of 28 students participated in the experiment, 24 (85.7%) of whom identified as female and 4 (14.3%) as male; no participants identified as nonbinary. Their ages ranged from 19 to 31 years (mean 23.4, SD 2.9 years). Of the 28 participants, 26 (92.9%) studied human medicine and 2 (7.1%) studied midwifery. The semesters varied from the second to the tenth semester, and 1 (3.6%) participant was in their final year. No participant was excluded from the analysis.

### Conversation Length and Part of Conversation

**Figure 2:**
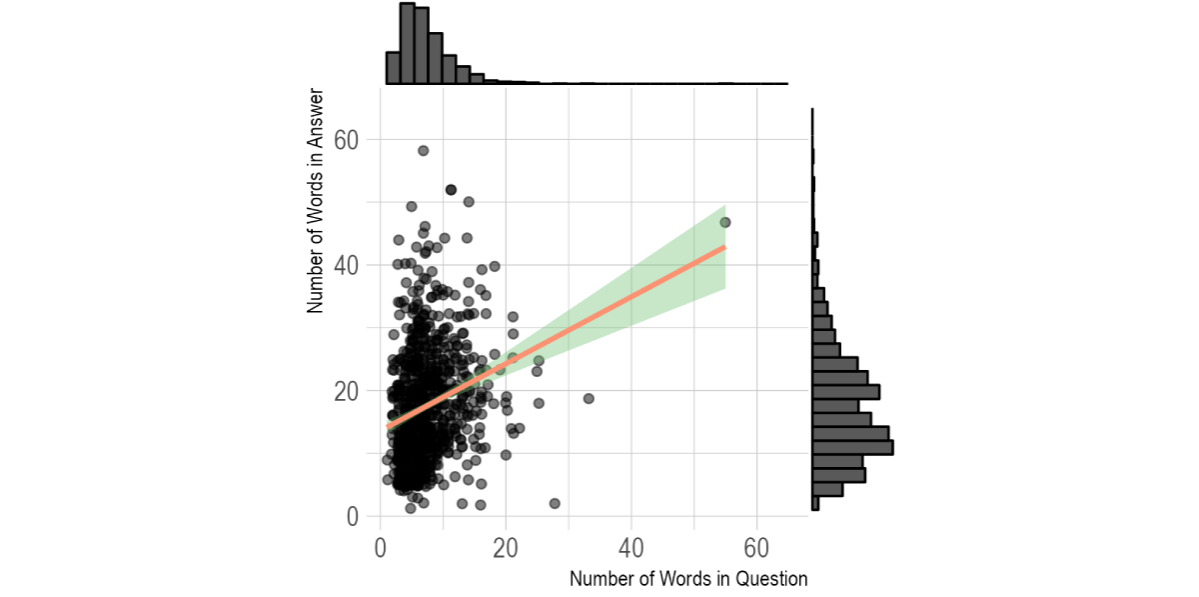
Scatter plot including the trend line for the number of words in the student’s question (x axis) and the number of words in the GPT answer (y axis). Representative variables are displayed as histograms at the top and along the right side. GPT: generative pretrained transformer.

A total of 28 conversations yielded 826 QAPs. Each conversation consisted of a median of 27.5 QAPs (Q25-Q75: 19.8-36.5 QAPs). The questions asked by participants yielded a median of 6 words (Q25-Q75: 6-9 words). The answers provided by GPT had a median of 16 words (Q25-Q75: 11-23 words). The Spearman correlation coefficient between the word count of the question and the word count of the answer was significant (*P*<.01), with ρ=0.29, indicating a positive but mild correlation. A scatter plot is displayed in Figure 2.

Of the 826 QAPs, most were related to history taking (n=782, 94.7%). A minority reflected interactive exchange (n=17, 2.1%), greeting/introduction (n=15, 1.8%), and goodbye/outlook (n=12, 1.6%).

### Content Analysis of Conversations

#### How Do Questions and Answers Relate in the Context of the Script?

**Figure 3:**
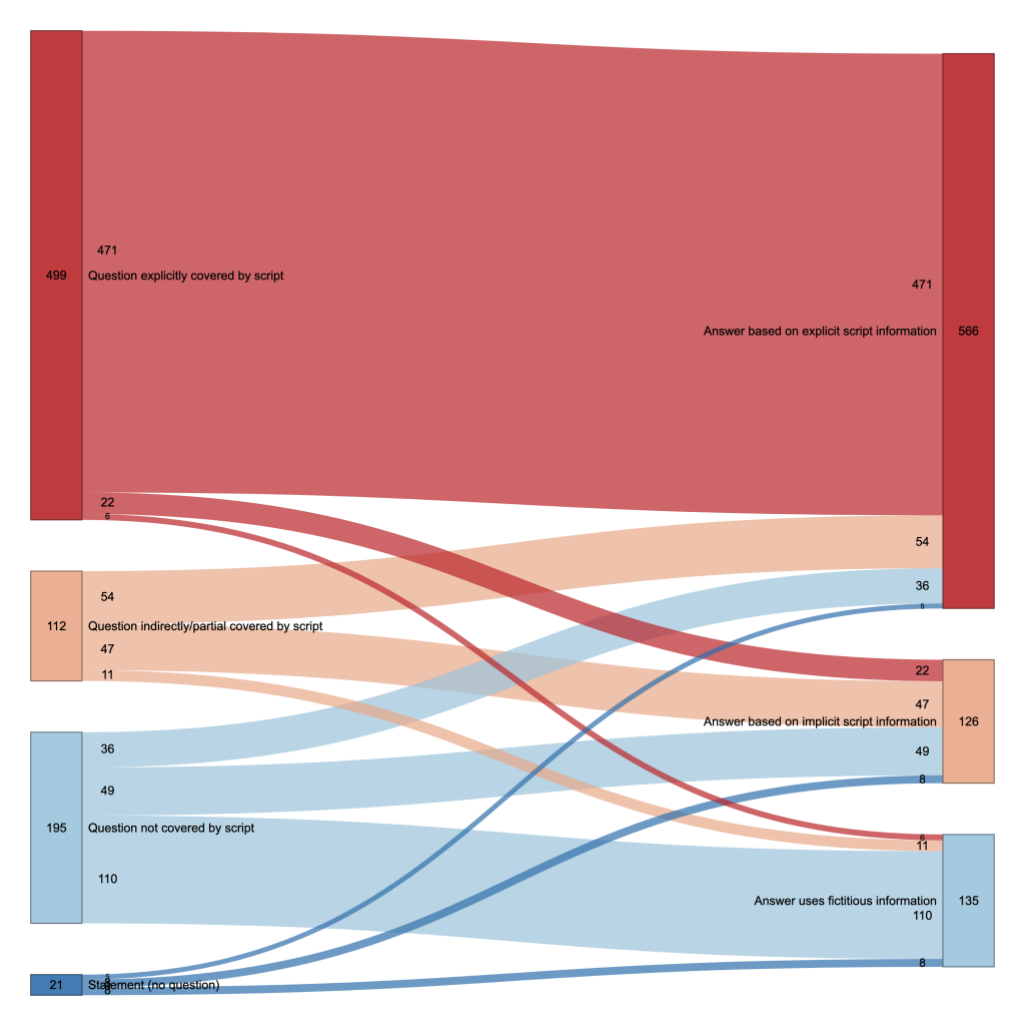
Sankey plot for “Student’s question covered by the illness script” and “GPT’s answer uses the illness script” categories in relationship to one another. Numbers indicate the total QAPs per group or connection, and connections without numbers are 0. GPT: generative pretrained transformer; QAP: question-answer pair.

In the subsequent assessment, we examined whether the questions posed by the students were covered by the script. We then analyzed how the GPT responses were based on the information provided in the script (Figure 3).

For questions explicitly covered by the script (n=502, 60.3%), 471 (94.4%) of GPT’s answers were based on explicit script information, 22 (4.4%) on implicit script information, and 6 (1.2%) on fictitious information. When the questions were indirectly or partially covered by the script (n=112, 13.4%), 54 (48.2%) of GPT’s responses were based on explicit information, 47 (42%) on implicit information, and 11 (9.8%) on fictitious information. For questions not covered by the script (n=195, 23.4%), 36 (18.5%) of GPT’s answers used explicit script information, 49 (25.1%) used implicit script information, and 110 (56.4%) used fictitious information. In instances where students provided statements without posing questions (n=24, 2.9%), 5 (23.8%) of GPT’s responses were based on the explicit script, 8 (38.1%) on the implicit script, and 8 (38.1%) on fictitious information. A total of 33 (3.8%) QAPs were excluded, because they could not be assessed in 1 of the 2 evaluated categories.

#### Are the GPT Answers Plausible?

When analyzing the answers in detail, 33 (4%) of the 826 QAPs concerned multiple aspects (ie, related to different questions or multiple parts of the illness script). We consequently further divided 32 (97%) QAPs into 2 QAPs and 1 (3%) QAP into 3 QAPs. In total, this resulted in 860 QAPs that were used for the subsequent qualitative plausibility analysis.

We further analyzed whether the GPT-provided responses were medically plausible. Of the 860 QAPs, 842 (97.9%) were rated as plausible. Specifically, 264 (30.7%) were rated as “answer plausible, highly specific for case,” 252 (29.3%) as “answer plausible, relevant for case,” and 326 (37.9%) as “answer plausible, not case specific.” A smaller proportion (n=14, 1.6%) were rated as rather implausible, while 2 (0.2%) were found to be very implausible. This rating could not be applied to 2 (0.2%) QAPs.

#### Correlation Between Reliance on the Illness Script and Plausibility

**Figure 4:**
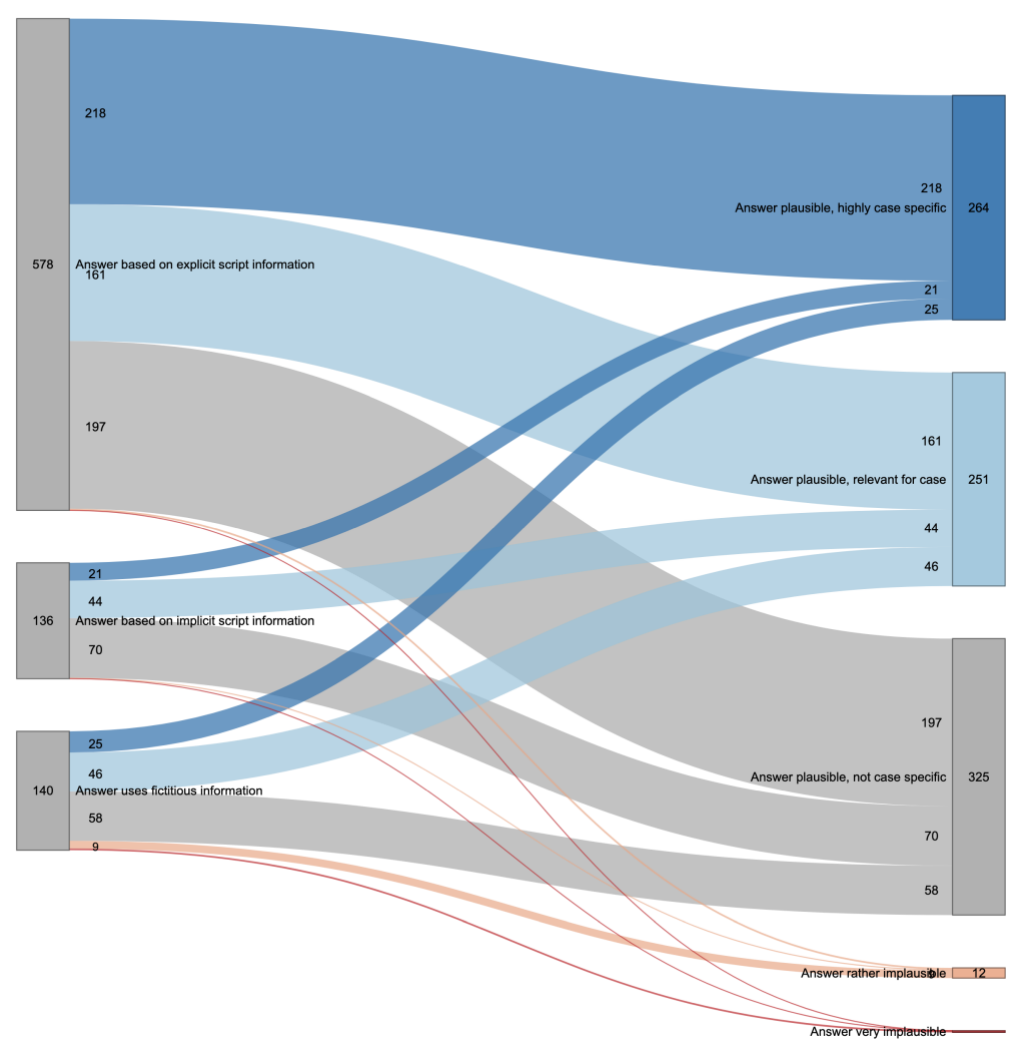
Sankey plot for “GPT’s answer uses the illness script” and answer plausibility categories in relationship to one another. Numbers indicate the total QAPs per group or connection, and connections without numbers are 0. GPT: generative pretrained transformer; QAP: question-answer pair.

We further analyzed whether the answers used explicit or implicit information from the illness script or fictitious information (Figure 4).

Among answers that used explicit script information (n=578, 67.7%), 218 (37.7%) were “plausible, highly specific for the case,” 161 (27.9%) were “plausible, relevant for the case,” and 197 (34.1%) were “plausible, not case specific,” with a mere 2 (0.3%) answers being rather implausible and none very implausible.

Among answers stemming from implicit script information (n=136, 15.9%), 21 (15.4%) were “plausible, highly specific for the case,” 44 (32.4%) were “plausible, relevant for the case,” and the majority (n=70, 51.5%) were “plausible, not case specific.” Only 1 (0.7%) answer was deemed rather implausible, and none were rated as very implausible.

In the context of fictitious information (n=140, 16.4%), the answers were varied: 25 (17.9%) were “plausible, highly specific for the case,” 46 (32.9%) were “plausible, relevant for the case,” and 58 (41.4%) were “plausible, not case specific.” Additionally, 9 (6.4%) answers rated as were rather implausible, and 2 (1.4%) were viewed as very implausible.

Furthermore, 6 (0.7%) answers could not be categorized.

#### Analysis of Implausible Answers

Finally, we analyzed all answers rated as rather or very implausible. Of the 14 (2.1%) answers that were rated as rather implausible, 7 (50%) were rated as socially desirable. A recurrent example for this category could be observed when the GPT-powered chatbot was asked for its eating habits; in these cases, the answers contained popular eating recommendations, instead of eating habits that were plausible for our case. For another 2 (14.3%) answers, the model did not stick to its rule as a simulated patient but tried to assist the user (ie, when greeted, the simulated patient asked the doctor, “How can I help you?”). For 1 (7.1%) other QAP, the model referred to the doctor by the name of the patient, which thus rated this QAP as “GPT leaving its role identity.” In another case, information clearly evident from the script (ie, vertigo) was not used and the simulated patient stated that he did not suffer from vertigo. One more rather implausible QAP was illogical in itself (ie, “But due to my weight loss, I have had a reduced appetite lately.”).

Among the 2 (0.2%) QAPs rated as very implausible, GPT escaped its role in 1 (50%) case. Herein, the participant asked about what can be seen in the physical exam, and the GPT-provided answer was, “Sorry, I am a language AI and do not have access to visual information. I can only provide information that is given to me through text input. Please consult a doctor for a complete clinical examination.” The second QAP was rated as very implausible due to a calculation error by GPT: When our chatbot was asked how much he drinks during 1 week, the answer was 10-12 L. Our script indicated 4-5 L per day, however, which would be an average of 28-35 L per week.

### Chatbot Usability Questionnaire

**Figure 5:**
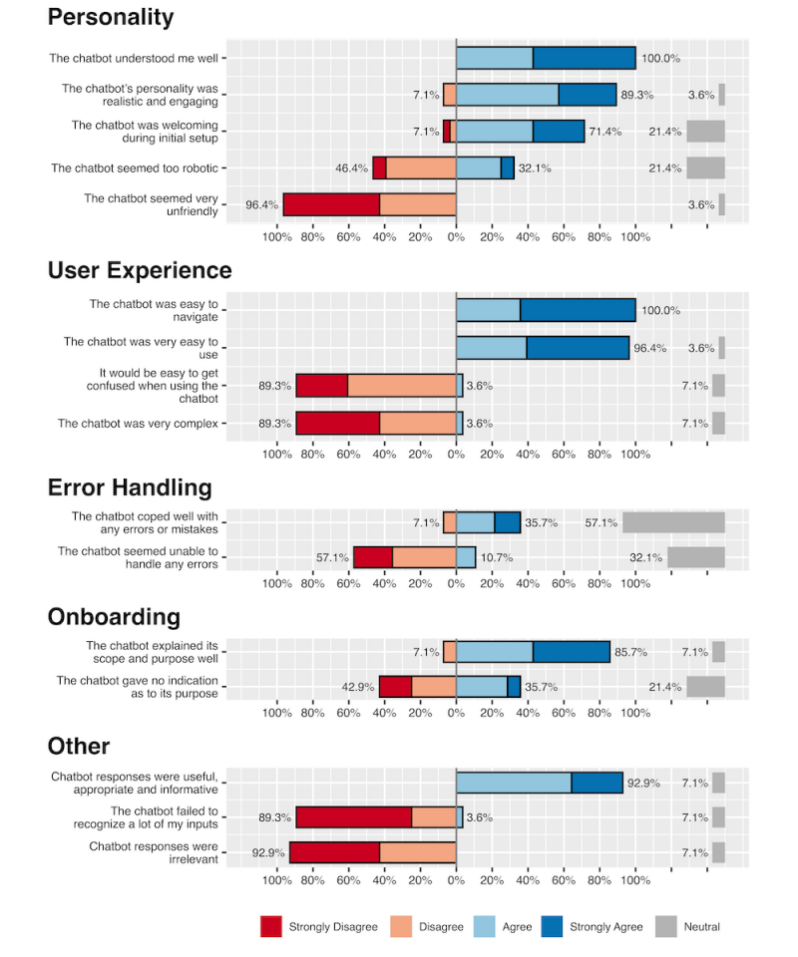
Results of the CUQ, grouped by category, as proposed by Holmes et al. 2023. Neutral responses are indicated on the right side of the figure. CUQ: Chatbot Usability Questionnaire.

The results of the CUQ are displayed in Figure 5 (also see Multimedia Appendix 2 for numeric results).

Within the personality category, the majority of respondents (n=16, 57%) felt the chatbot’s personality was realistic and engaging and 9 (32%) strongly agreed. When considering whether the chatbot seemed too robotic, a large proportion (n=13, 46%) disagreed and 2 (7%) strongly disagreed. The chatbot was perceived as welcoming during the initial setup by 12 (43%) of respondents, and 8 (29%) respondents strongly agreed. A significant portion (n=15, 54%) strongly disagreed, and 12 (43%) disagreed with the notion that the chatbot seemed unfriendly. In terms of understanding, 12 (43%) respondents agreed and 16 (57%) strongly agreed that the chatbot understood them well.

For the user experience category, the chatbot was seen as easy to navigate by 10 (36%) respondents, with a notable 18 (64%) strongly agreeing. In contrast, when asked whether it would be easy to get confused when using the chatbot, 17 (61%) disagreed and 8 (29%) strongly disagreed. The chatbot’s ease of use was highlighted by 11 (39%) respondents agreeing and 16 (57%) strongly agreeing. Most respondents disagreed with the perception that the chatbot was complex: 12 (43%) disagreed and 13 (46%) strongly disagreed.

In the error handling category, a majority (n=16, 57%) of the respondents remained neutral about the chatbot coping well with errors. Of the remainder, most respondents were positive about the error handling, with 6 (21%) agreeing and 4 (14%) strongly agreeing. Conversely, 6 (21%) respondents strongly disagreed and 10 (36%) disagreed that the chatbot seemed unable to handle errors, with only a minority (n=3, 11%) agreeing.

For the onboarding category, 12 (43%) respondents agreed and another 12 (43%) strongly agreed that the chatbot explained its scope and purpose well. Accordingly, 8 (29%) respondents agreed, 7 (25%) disagreed, and 5 (18%) strongly disagreed with the statement that the chatbot gave no indication as to its purpose.

For questions not related to a factor, 18 (64%) respondents agreed and 8 (29%) strongly agreed that chatbot responses were useful, appropriate, and informative. Accordingly, 14 (50%) respondents strongly disagreed and 12 (43%) disagreed that chatbot responses were irrelevant. Additionally, 18 (64%) respondents strongly disagreed and 7 (25%) disagreed with the statement that the chatbot failed to recognize many inputs.

Overall, the CUQ score was 77 (Q25-Q75: 71-83) out of a maximum score of 100, which indicated a positive user experience with the chatbot.

### Improved AI-Capable Illness Script

Finally, we analyzed the QAPs for aspects on how to improve the illness script. Of 302 QAPs where the student’s question was either not covered or only indirectly/partially covered by the script, we were able to further classify 301 (99.7%) QAPs as to whether the script needs to be updated. The 1 (0.3%) unclassified QAP consisted of an uncontextual exchange and was thus discarded.

#### QAPs Implicating an Update of the Illness Script

For the majority of the QAPs (n=141, 46.8%), no update was required, as the information was not relevant for the case, although it was medically relevant. A further 14 (4.7%) QAPs were neither medically relevant nor relevant for the case, also not implicating an update. For 86 (28.6%) QAPs, however, we determined that an already existing criterion in our illness script needed further details. Moreover, for 60 (19.9%) of the analyzed QAPs, we judged that our illness script needed additional criteria.

#### Detailed Additions to Existing Criteria

More detailed specifications were recommended for some of the already existing criteria. These encompassed the specification of vomiting, nausea, stress, daily symptom progression, timing of individual symptoms throughout the day, attempts at relief, prior investigations, urine output, bedding/nightclothes, and stool.

#### Specific New Criteria Required

A closer examination of the content revealed several specific criteria that were absent but found to be relevant. These included dietary habits, activity/sports, pain, travel abroad, urine, and potential autoimmune diseases.

#### Improved Script Version

Based on the aforementioned information, we generated an updated version of our illness script (Multimedia Appendix 3).

## Discussion

### Principal Findings

In this study, we investigated the capabilities of GPT used as a chatbot to practice history taking, a core competency of medical professionals [1,2]. Using a mixed methods approach, we provided a comprehensive overview of the performance of GPT, as well as the perception of our participants about the chatbot. Our main findings can be divided into 2 areas: the performance of GPT as a simulated patient and how medical students perceive this chatbot as a conversational agent.

#### Performance of GPT as a Simulated Patient

When developing our chatbot, our focus was the feasibility of using an LLM model as a simulated patient. Before incorporation of our chatbot, we developed a prompt consisting of behavioral instructions and a chatbot-optimized illness script. Our analysis revealed that GPT was capable of providing most of the answers that were medically plausible and in line with the illness script. When questions were covered by the script, GPT was capable of referring to them, even when the information was only present in an implicit form (Figure 3). Even if questions were not covered by the script, GPT used the information from our medical case to generate answers that were mostly medically plausible. However, our analysis revealed that the degree of plausibility decreased when less information was present in the script (Figure 4).

The ability of GPT to act as a simulated patient requires reasoning capabilities (ie, thinking about something in a logical and systematic way) [40-45]. There are different types of scientifically recognized reasoning, such as deductive reasoning that applies a general rule to a specific case, inductive reasoning that uses specific observations to draw a general rule, and abductive reasoning that finds the best conclusion for some observations [40]. Although LLMs, such as GPT, have been successful in various reasoning areas [46], our investigation revealed some caveats.

As most of the GPT answers were based on explicit script information, providing the user with these details did not necessitate the generation of new ideas and was thus a mere task of reformulating the given information for the context of a conversation. As a LLM [29], it was not surprising that GPT mastered this task. Regarding information that is not or only indirectly evident from the script, however, we postulated that both abductive and commonsense reasoning capabilities would be required; for these answers, we observed more implausible answers when compared to answers that were based on explicit script information.

Indeed, GPT-3.5 is known to perform reasonably well in both abductive and commonsense reasoning tasks [46,47]; our data confirmed these observations. There were a few instances when GPT provided implausible responses, however, and our content analysis revealed a tendency toward socially desirable answers. These errors could be interpreted as “escaping” abductive reasoning and applying deductive reasoning instead, thereby using general principles (eg, about a healthy diet) for a specific case. A similar observation was made by Espejel et al [46], when GPT “ignored” provided information and instead “relies on its general knowledge and understanding of the world.”

Regarding our illness script, these examples highlight that the illness script must include details about the patient role, especially when the patient displays traits that do not match popular or socially accepted norms. Although our script was capable of providing most information required for history taking either explicitly or implicitly, some criteria missed important details, while other criteria were completely missing. With the intention of keeping the illness script as short as possible and thereby reduce the work for teachers, we used the data from our study to amend our illness script.

Of note, we found a positive correlation between the word count of the question and the word count of the answer of GPT. Although the correlation was rather mild, possible interpretations for this behavior include GPT mimicking the language style (and length) of the interview, as well as inputs containing multiple questions, thus provoking longer answers. Although our analysis does not provide insight into this question, our data imply that future prompts should focus more on specifying the conversation style of GPT to achieve a standardized patient experience.

#### Perception of Medical Students

After exploring the performance of GPT as a simulated patient, we interviewed our participants about their perceptions of our chatbot using the CUQ. Confirming the qualitative analysis we performed, the students rated our chatbot as realistic and engaging. Again, in line with our qualitative data, the chatbot was rated as useful, appropriate, and relevant, with only a negligible number of students stating that the chatbot did not recognize their inputs; notably, some issues were detected with our chatbot being robotic. These data largely confirm the linguistic capabilities of GPT-3.5, with its output even showing personality traits [48-51]. Given the importance of the chatbot’s authenticity to provide students with a plausible conversation partner to practice their skills, the results of the CUQ are reassuring that GPT is capable of providing this experience.

### Comparison With Prior Work

Owing to the costs and potential disturbances associated with the use of real or simulated patients in communication training [52,53], there has been great interest in the use of virtual simulated patients as chatbots for communication training [21,31]. In the past years, studies were published using chatbots to cover a wide range of conditions and domains [52,53]. In addition to physician-patient communication skills, chatbots have been used for interprofessional communication [54] and for skill assessments [55]. However, in contrast to our study, most of these studies were performed before the broad accessibility of LLMs, such as GPT. These chatbots have thus been restricted in their authentic skills, capability of adoption (ie, in terms of personality, cases, etc), and ability to be transferred to different health care domains [31]. Although we also focused on 1 patient case, the ability of LLMs makes them theoretically capable of adapting to a given situation. Furthermore, our assessment using the CUQ revealed that our chatbot was perceived as realistic. This indicates that LLMs, such as GPT, when investigated rigorously, might be able to overcome the aforementioned restrictions.

As is the case with the technology used to process and generate language, previous studies have used various interfaces [52,53]. Similar to our study, many rely on web-based chat-like interfaces, and good usability seems to be of importance for acceptance by the learners [56]. Indeed, the CUQ used in our study also revealed that our user interface yields a good user experience. However, even with good acceptance, chat-like interfaces are limited to written language, thus restricting communication to the verbal domain. Therefore, newer approaches integrate chatbots in virtual reality environments [54], paving the way for a more integrated learning experience.

### Limitations

Our study has some noteworthy limitations. As this was the first study using GPT as a simulated patient, we focused on 1 language model (ie, GPT-3.5, which we chose for its free availability and fast response time) and 1 patient case. Although we perceived our case as representative for history taking, our data did not allow for generalization to more specialized medical fields, and further studies are required to verify scalability to other medical specialties. Moreover, we focused on history taking, and although our chatbot performed well in general communication skills, it remains unclear how it will perform in other areas. Additionally, history taking is usually performed with spoken language, in contrast to the written language we used in our investigation. As this was a feasibility study, we only interviewed our participants about their perceptions but did not perform any objective skill measurements. We therefore cannot conclude that our participants improved in history taking, which should be addressed in future studies. Furthermore, the majority of our participants were female, which may have reduced the generalizability of our results. Due to the fact that we designed our study as an exploratory feasibility study, we did not perform a sample size calculation and therefore used descriptive statistics almost exclusively. Moreover, our participants were volunteers and thus probably motivated toward AI technology [22], possibly indicating a selection bias.

### Conclusion

This study showed that a GPT-powered simulated patient chatbot works well and is perceived favorably among medical students. Although real patients remain the cornerstone of clinical teaching, technology-based education, as shown in this study, could be particularly beneficial for novice learners during their initial learning phases. It is important to note that we did not investigate skill acquisition, which is an important next step when evaluating GPT-based chatbots. Furthermore, our chatbot could be combined with other new technologies, such as speech recognition and virtual/augmented reality, and thus could offer an even more integrated learning environment. Despite limitations, our study has implications for the field of medical education. Most importantly, we could show that GPT is capable of providing a simulated patient experience using an illness script, paving the way toward technology-assisted acquisition of communication skills. Moreover, by showing the capabilities of GPT-3.5 in history taking, the technology of LLMs might be capable of assisting learners in other areas as well.

## Appendix

Multimedia Appendix 1 Full prompt.

Multimedia Appendix 2 CUQ results table. CUQ: Chatbot Usability Questionnaire.

Multimedia Appendix 3 Illness script.

## Abbreviations

AI: artificial intelligence
API: application programming interface
CUQ: Chatbot Usability Questionnaire
GPT: generative pretrained transformer
LLM: large language model
QAP: question-answer pair

## Data Availability

The data sets used and analyzed during this study are available from the corresponding author upon reasonable request.
