# Supplementary material for: A Generative Pretrained Transformer (GPT)–Powered Chatbot as a Simulated Patient to Practice History Taking: Prospective, Mixed Methods Study
Source: JMIR Med Educ. 2024 Jan 16;10:e53961. doi: 10.2196/53961 (PMC10828948; doi:10.2196/53961)
Supplement: Multimedia Appendix 2 [file mededu_v10i1e53961_app2.pdf]

Multimedia Appendix 2: CUQ results table

Results of the Chatbot Usability Questionnaire by question. Results are indicated as percentage and absolute numbers in brackets (i.e., % [n]).

Item	Strongly Disagree	Disagree	Neutral	Agree	Strongly Agree
The chatbot’s personality was realistic and engaging	0% (0)	7.1% (2)	3.6% (1)	57.1% (16)	32.1% (9)
The chatbot seemed too robotic	7.1% (2)	39.3% (11)	21.4% (6)	25% (7)	7.1% (2)
The chatbot was welcoming during initial setup	3.6% (1)	3.6% (1)	21.4% (6)	42.9% (12)	28.6% (8)
The chatbot seemed very unfriendly	53.6% (15)	42.9% (12)	3.6% (1)	0% (0)	0% (0)
The chatbot explained its scope and purpose well	0% (0)	7.1% (2)	7.1% (2)	42.9% (12)	42.9% (12)
The chatbot gave no indication as to its purpose	17.9% (5)	25% (7)	21.4% (6)	28.6% (8)	7.1% (2)
The chatbot was easy to navigate	0% (0)	0% (0)	0% (0)	35.7% (10)	64.3% (18)
It would be easy to get confused when using the chatbot	28.6% (8)	60.7% (17)	7.1% (2)	3.6% (1)	0% (0)
The chatbot understood me well	0% (0)	0% (0)	0% (0)	42.9% (12)	57.1% (16)
The chatbot failed to recognise a lot of my inputs	64.3% (18)	25% (7)	7.1% (2)	3.6% (1)	0% (0)
Chatbot responses were useful, appropriate and informative	0% (0)	0% (0)	7.1% (2)	64.3% (18)	28.6% (8)
Chatbot responses were irrelevant	50% (14)	42.9% (12)	7.1% (2)	0% (0)	0% (0)
The chatbot coped well with any errors or mistakes	0% (0)	7.1% (2)	57.1% (16)	21.4% (6)	14.3% (4)

Item	Strongly Disagree	Disagree	Neutral	Agree	Strongly Agree
The chatbot seemed unable to handle any errors	21.4% (6)	35.7% (10)	32.1% (9)	10.7% (3)	0% (0)
The chatbot was very easy to use	0% (0)	0% (0)	3.6% (1)	39.3% (11)	57.1% (16)
The chatbot was very complex	46.4% (13)	42.9% (12)	7.1% (2)	3.6% (1)	0% (0)
