# Supplementary material for: A Generative Pretrained Transformer (GPT)–Powered Chatbot as a Simulated Patient to Practice History Taking: Prospective, Mixed Methods Study
Source: JMIR Med Educ. 2024 Jan 16;10:e53961. doi: 10.2196/53961 (PMC10828948; doi:10.2196/53961)
Supplement: Multimedia Appendix 3 [file mededu_v10i1e53961_app3.pdf]

**Course of study:**	**Medicine**
**Departement:**	**Internal Medicine**
Learning objective:	
• didactic:	
• medical:	
Prior knowledge:	

Main symptom: „Leading symptom“

**Sector 1:**

- Character/presentation

Your name is XXX, you are XXX years old, and by profession, you are XXX. Currently, you feel XXX.

- Background Information
  - Family and social integration
  - Professional context
  - Stress?
  - Pertinent issues for the case, such as distressing topics (e.g., separation, unsuccessful diet attempts, …).
  - Medical history, including mentioning the current care team (general practitioner, etc.)
  - Preliminary examinations/previous findings (including preventive care) especially in relation to the current reason for presentation. (Also, negative statements (e.g., "…no prior examinations have been done…").
  - …

A detailed description, especially of the social and emotional background, is recommended. If not mentioned, GPT often chooses the "most likely" (e.g. wife and 2 children) or the socially desirable (e.g. “I eat healthily and variably”). Conversely, this means socially undesirable topics should be explicitly mentioned in the script.
Overall, it's better to have an extra sentence for character presentation so that ChatGPT can refer to it for questions that might not be covered in the script.

- Setting:
  Setting (delete what does not apply):
  General practitioner's office
  Hospital

Nursing home
Home visit
Video consultation

Details about the type of presentation (initial consultation, follow-up, emergency, ...)

- Medical History:
    - Detailed description of the current situation with physical and psychological symptoms.
    - Preliminary examinations/previous findings (including preventive care).
    - Mention the trigger for the current presentation if necessary.
    - Mention prior attempts at relief.
    - Personal illness theory? Tried any treatments? Have other doctors been consulted?

It is advisable to describe the symptoms mentioned under sections 2 and 3 in detail to minimize any deviations by ChatGPT.
In this context, it's especially important to mention clues that are relevant for differential diagnosis (for example, 'slightly increased sweating' versus 'changing pyjamas and bed linens nightly due to pronounced B-symptoms').

Further details are for example:

- *Timing of the onset of individual symptoms throughout the day, if relevant.*
- *Triggers for individual symptoms, if relevant.*
- *Effects of individual symptoms, if relevant.*

Sector 2:<br>Primary symptom and current medical history	
Main complaint, if applicable, with…	
Location	
Quality	
Severity	
Temporal occurrence	
Aggravation or alleviation of the symptom	
Triggering factors	
Accompanying symptoms	
Level of disability	
Previous illnesses related to the primary symptom	

Sector 3: Vegetative history	
Appetite	
Vomiting/nausea	
Thirst	
Intolerances	
Weight	
Cough	
Sputum	
Bowel movements	

Urine	
Fever	
Chills	
Night sweats	
Sleep	
Sexual history	
Pain	

Sector 4: System history/pre-existing conditions	
Nerves	
Sensory organs and psyche	
Cardiovascular diseases	
Lungs/bronchi	
Kidneys	
Stomach/intestines	
Liver/gallbladder	
Metabolism	
Blood disorders	
Rheumatism	
Allergies	
Autoimmune diseases	
Malignant diseases	
Infections	
Vaccinations	
Surgeries	
Accidents	
Travel abroad	

Sector 5:<br>Medications, Family history, Substances of abuse, Social history	
Current and past medication	
Dietary habits	
Physical activity/sports	
Alcohol	
Nicotine	
Drugs	
General practitioner	
Family history	
Occupation	

Sector 6:<br>Specialized medical history	e.g. gynecological/urological/psychiatric/… symptom complexes.
…	

Individual criteria may be unnecessary depending on the case/symptom constellation or may need to be added. Please incorporate additional criteria into the table matrix.
